# Supplementary material for: Spatial transcriptomics reveal markers of histopathological changes in Duchenne muscular dystrophy mouse models
Source: Nat Commun. 2023 Aug 15;14:4909. doi: 10.1038/s41467-023-40555-9 (PMC10427630; doi:10.1038/s41467-023-40555-9)
Supplement: Supplementary file 1 — Supplementary Information [file 41467_2023_40555_MOESM1_ESM.pdf]

Supplementary information

Spatial transcriptomics reveal markers of histopathological changes in Duchenne muscular dystrophy mouse models

L.G.M. Heezen, T. Abdelaal, M. van Putten, A. Aartsma-Rus, A. Mahfouz, P. Spitali
Corresponding author: Pietro Spitali, p.spitali@lumc.nl

In this supplementary information file:

Supplementary figures:

- Cluster marker genes per sample (Supplementary figures 1-4)
- Validation of identified neuromuscular junction cluster (Supplementary figures 5-6)
- Deconvolution results of all clusters (Supplementary figure 7)
- Comparison of regenerating myonuclei based on RNAscope gene expression vs. deconvolution results in *mdx* (Supplementary figure 8)
- Comparison of macrophages based on RNAscope gene expression vs. deconvolution results in D2-*mdx* (Supplementary figure 9)
- smFISH results on consecutive section of all Visium samples (Supplementary figures 10-13)
- Co-expression of identified regeneration markers in wildtype and mdx (Supplementary figure 14)
- smFISH results of independent samples as biological validation (Supplementary figure 15)
- RNAvelocity results on DBA/2J healthy background (Supplementary figure 16)

Supplementary tables:

- Imaging settings for neuromuscular junction staining (Supplementary Table 1)
- Product information smFISH HiPlex experiment (Supplementary Table 2)
- Imaging settings for smFISH experiments (Supplementary Table 3)

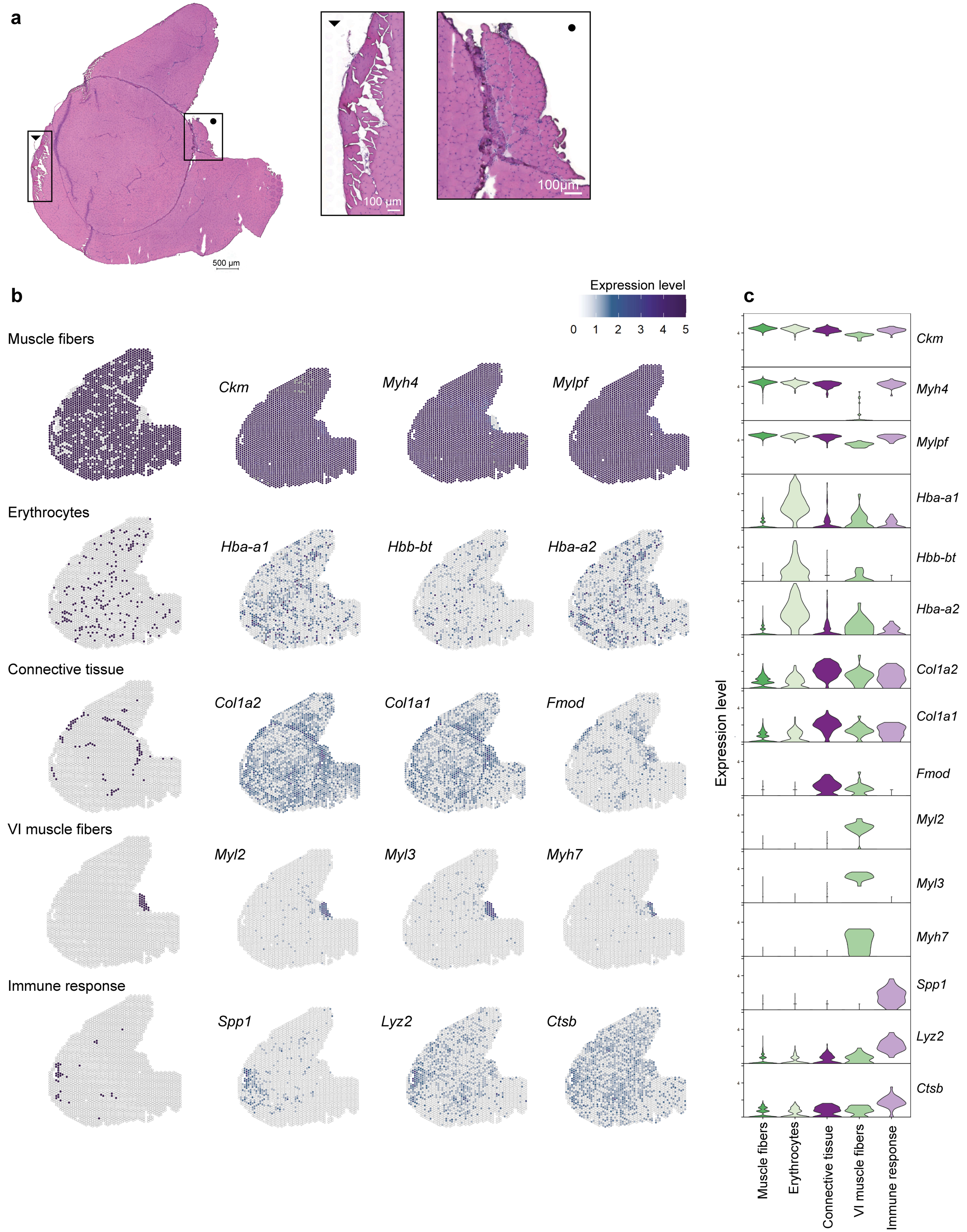

Supplementary Fig. 1. **Annotated clusters of the C57BL10 muscle based on histological features and the upregulated expression of marker genes in these clusters**. *(a) HE stained C57BL10 QUA sample with two zoomed-in areas, the triangle icon displaying damaged tissue area close to the immune response cluster, the dot icon showing VI muscle (b) All annotated clusters spatially plotted with the main marker genes and their gene expression level throughout the tissue section (c) Violin plot showing the expression level of the marker genes across the annotated clusters.*

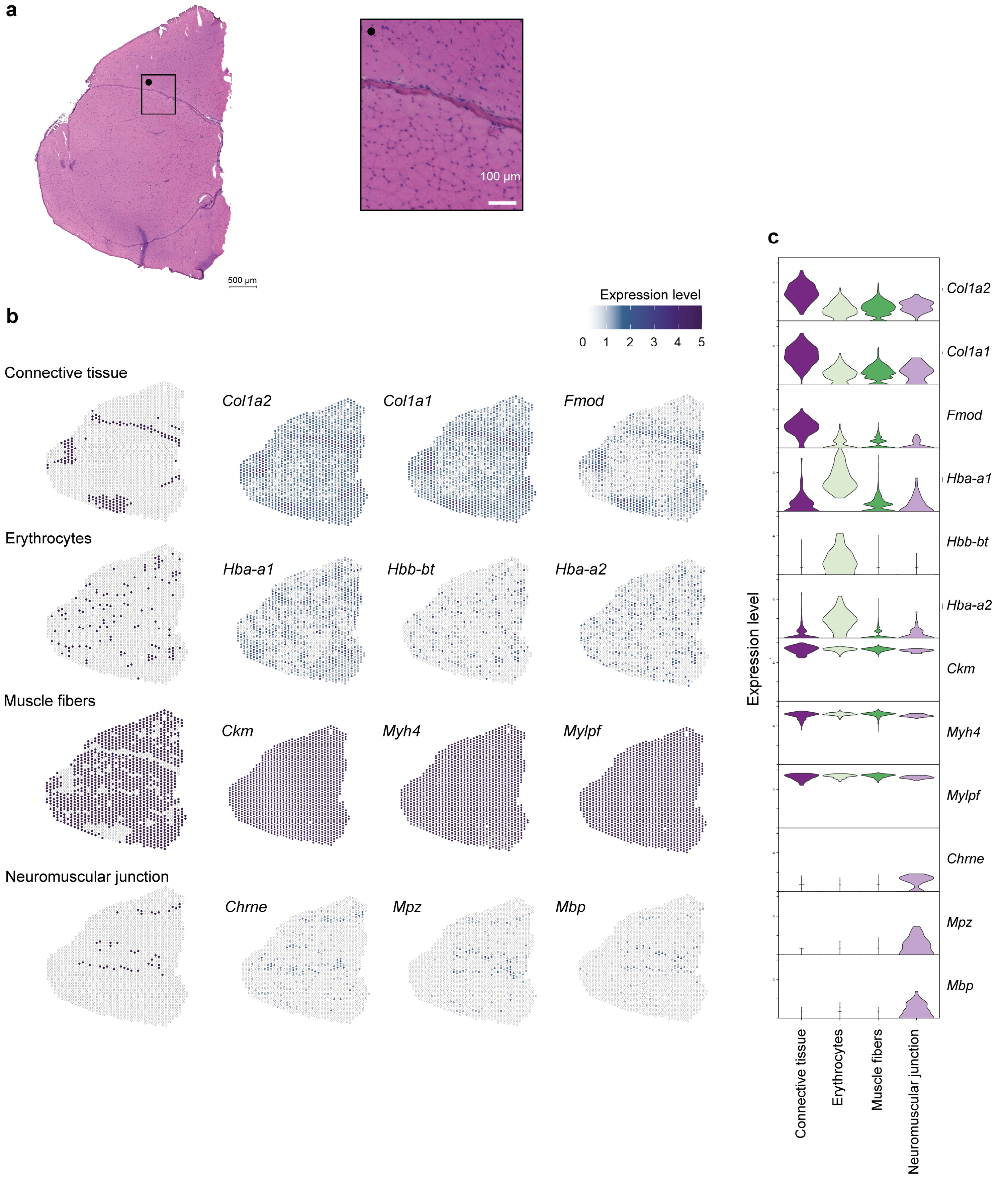

Supplementary Fig. 2. **Annotated clusters of the DBA/2J muscle based on histological features and the upregulated expression of marker genes in these clusters.** *(a) HE stained DBA/2J quadriceps sample with one zoomed-in area, the dot icon showing part of the perimysium (connective tissue sheet) and representative healthy muscle fibers. (b) All annotated clusters spatially plotted with the main marker genes and their gene expression level throughout the tissue section. (c) Violin plot showing the expression level of the marker genes across the annotated clusters.*

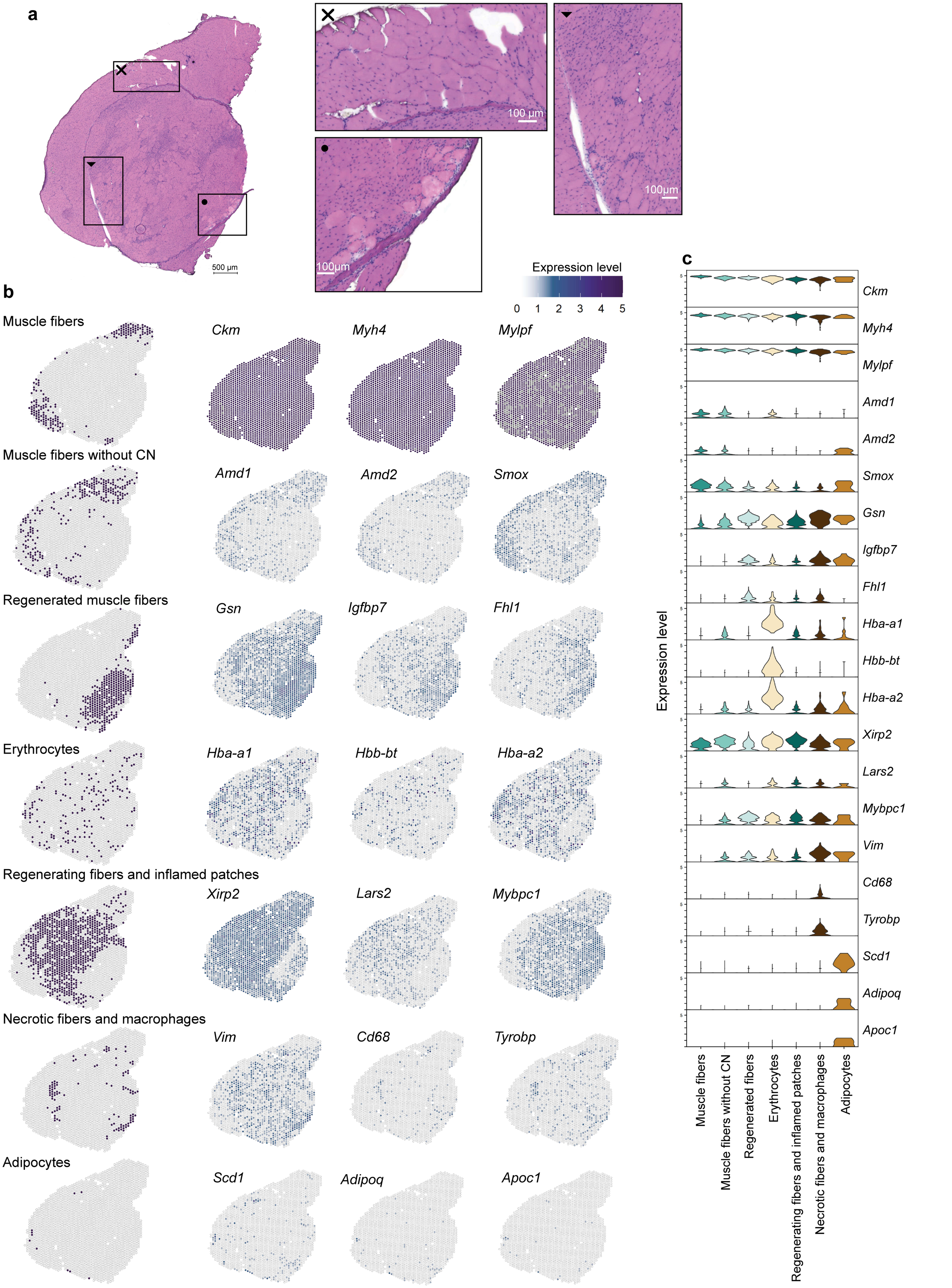

Supplementary Fig. 3. **Annotated clusters of the *mdx* muscle based on histological features and the upregulated expression of marker genes in these clusters.** *(a) HE stained mdx quadriceps sample with three zoomed-in areas, the cross icon displaying mature myofibers without central nuclei (CN), the dot icon showing some necrotic fibers and lastly, the triangle icon displaying an area of regeneration and mild inflammation. (b) All annotated clusters spatially plotted with the main marker genes and their gene expression level throughout the tissue section. (c) Violin plot showing the expression level of the marker genes across the annotated clusters.*

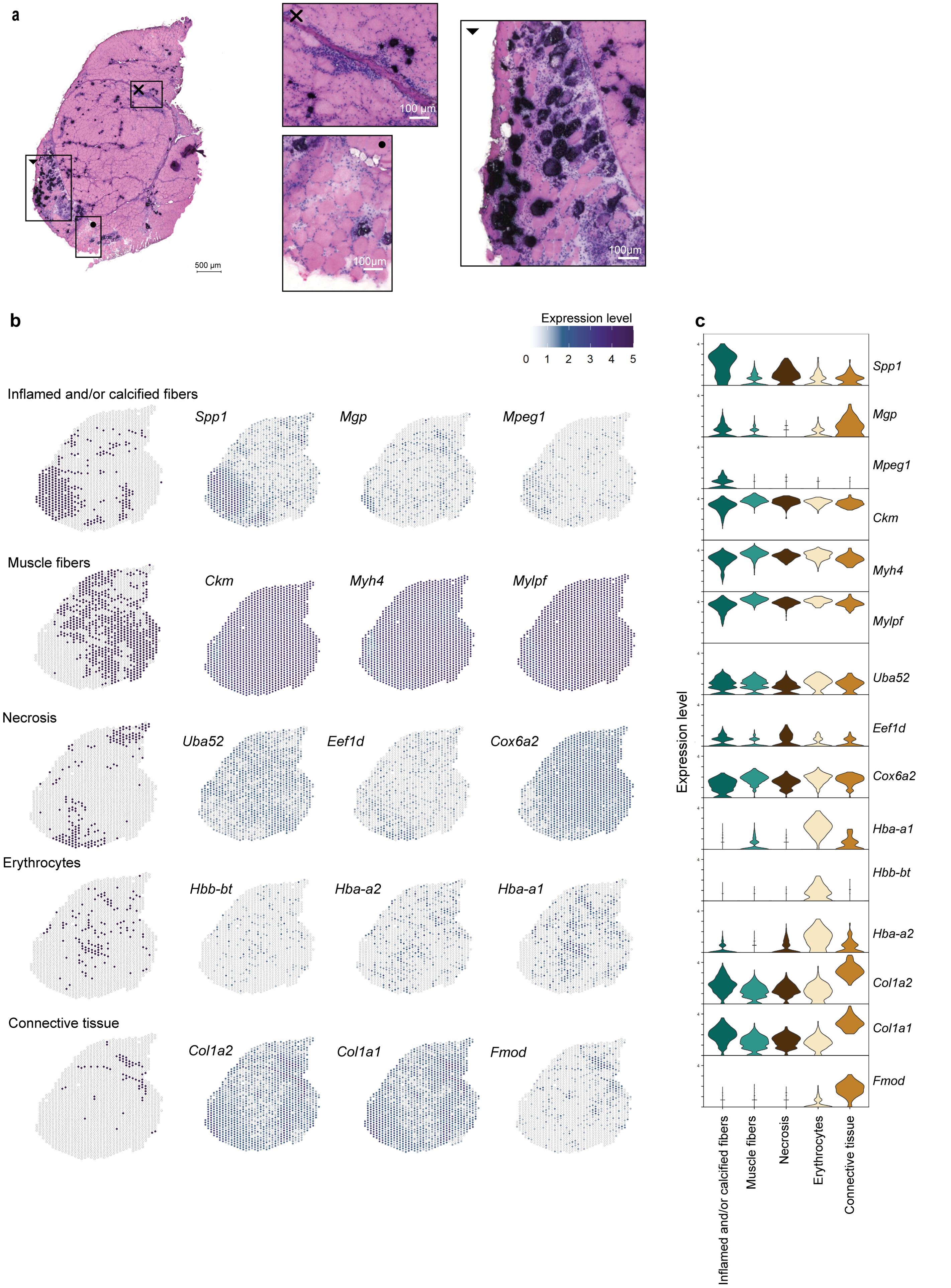

Supplementary Fig. 4. **D2-*mdx* annotated clusters based on histological features and the upregulated expression of marker genes in these clusters.** *(a) HE stained D2-mdx quadriceps sample with three zoomed-in areas, the cross icon displaying inflammation around the connective tissue sheet, the dot icon showing some necrotic fibers and lastly the triangle icon displaying the most severely affected area of the muscle section with extensive calcification, inflammation and fibrosis. (b) All annotated clusters spatially plotted with the main marker genes and their gene expression level throughout the tissue section. (c) Violin plot showing the expression level of the marker genes across the annotated clusters.*

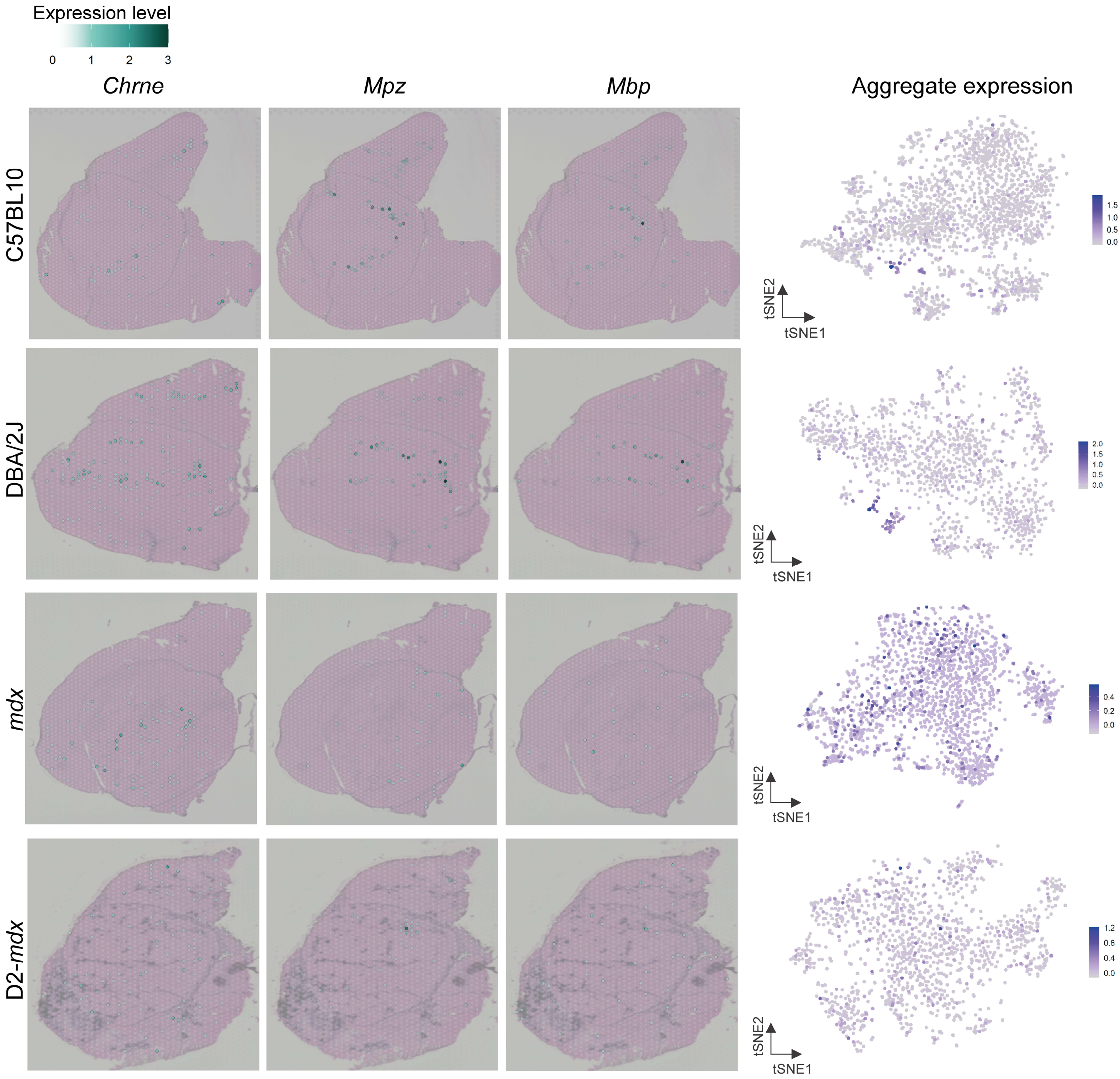

Supplementary Fig. 5. **Expression of NMJ marker genes.** *Expression of the NMJ marker genes (Mpz, Chrne and Mbp) identified in the DBA/2J sample plotted on the Visium images for all samples display clear patterning/expression in both wildtype samples, but decreased expression and no clear pattern in the Duchenne mouse models (mdx, D2-mdx). The aggregate expression of the NMJ marker genes is also plotted (far-right column) on the tSNE to reveal which spots reflect the NMJ expression profile. DBA/2J shows most distinct cluster, in C57BL10 one might also be able to pinpoint to a cluster of NMJ spots. However, for mdx and D2-mdx there is no clear clustering of aggregate expression.*

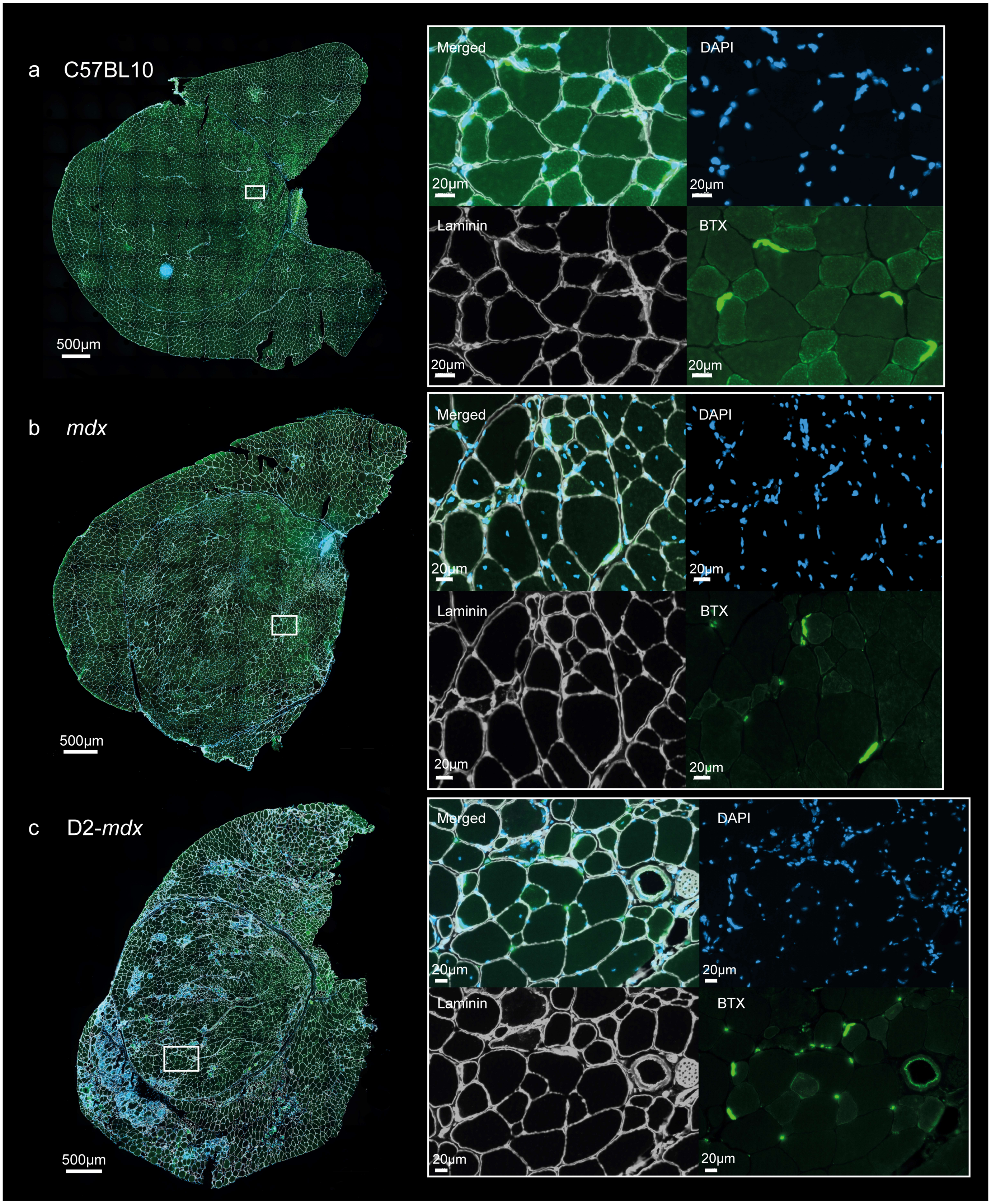

Supplementary Fig. 6. **BTX staining for NMJ consecutive section of the C57BL10, *mdx* and D2-*mdx* Visium samples.** *(a) BTX staining on C57BL10 reveals NMJs that seem to match to the location of the previously plotted NMJ marker genes (Figure S6). (b and c) The BTX staining on the mdx and D2-mdx sample reveals NMJs with altered, more fragmented structures which has previously been described as a characteristic of Duchenne mouse models.*

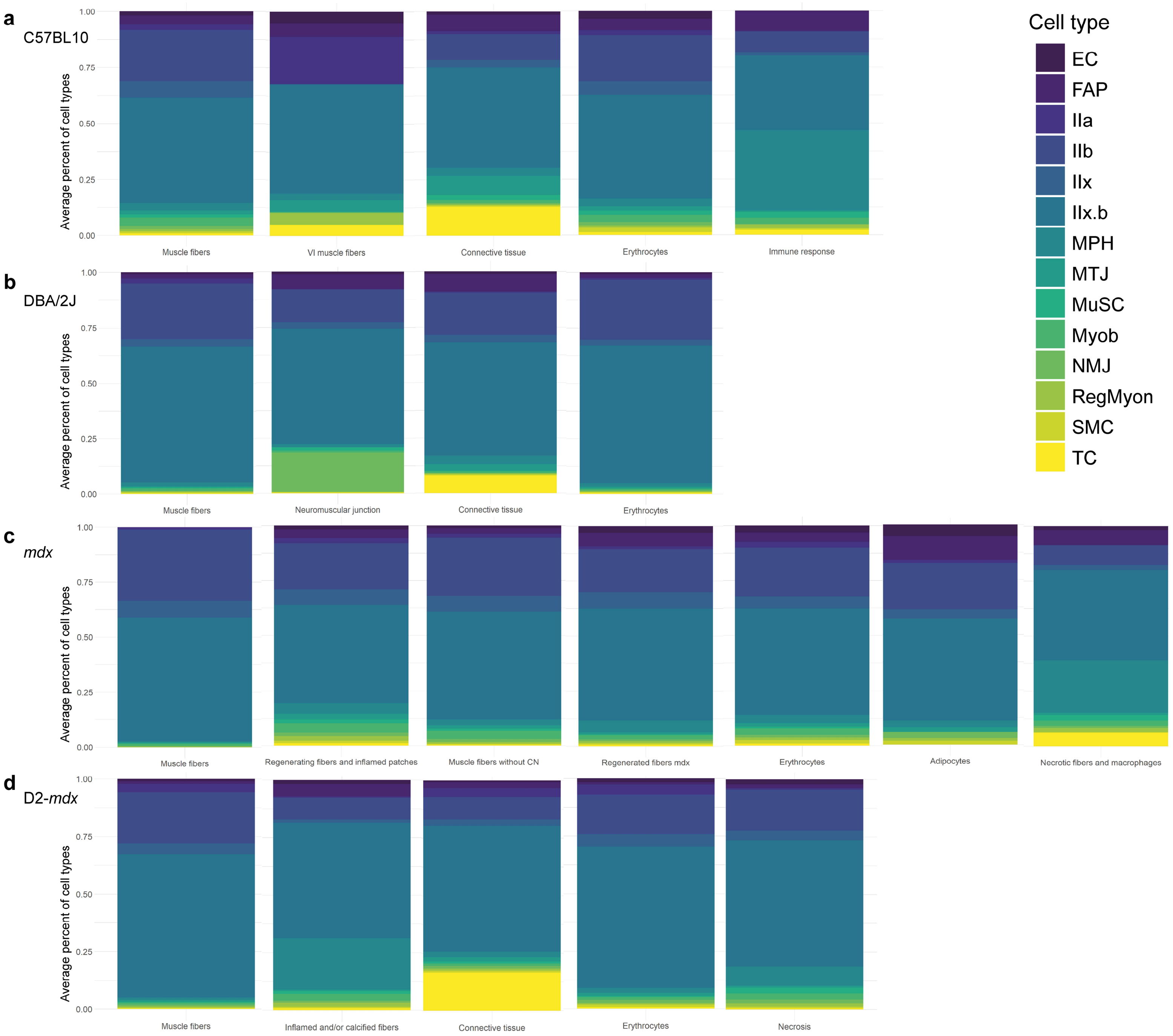

Supplementary Fig. 7. **Deconvolution of the spatial data using a snRNAseq reference dataset displays average percentage of contributing cell-types to annotated clusters.** *Stacked barplots displaying the average percentage of contributing cell-types to the annotated clusters for (a) C57BL10, (b) DBA/2J, (c) mdx and (d) D2-mdx.*

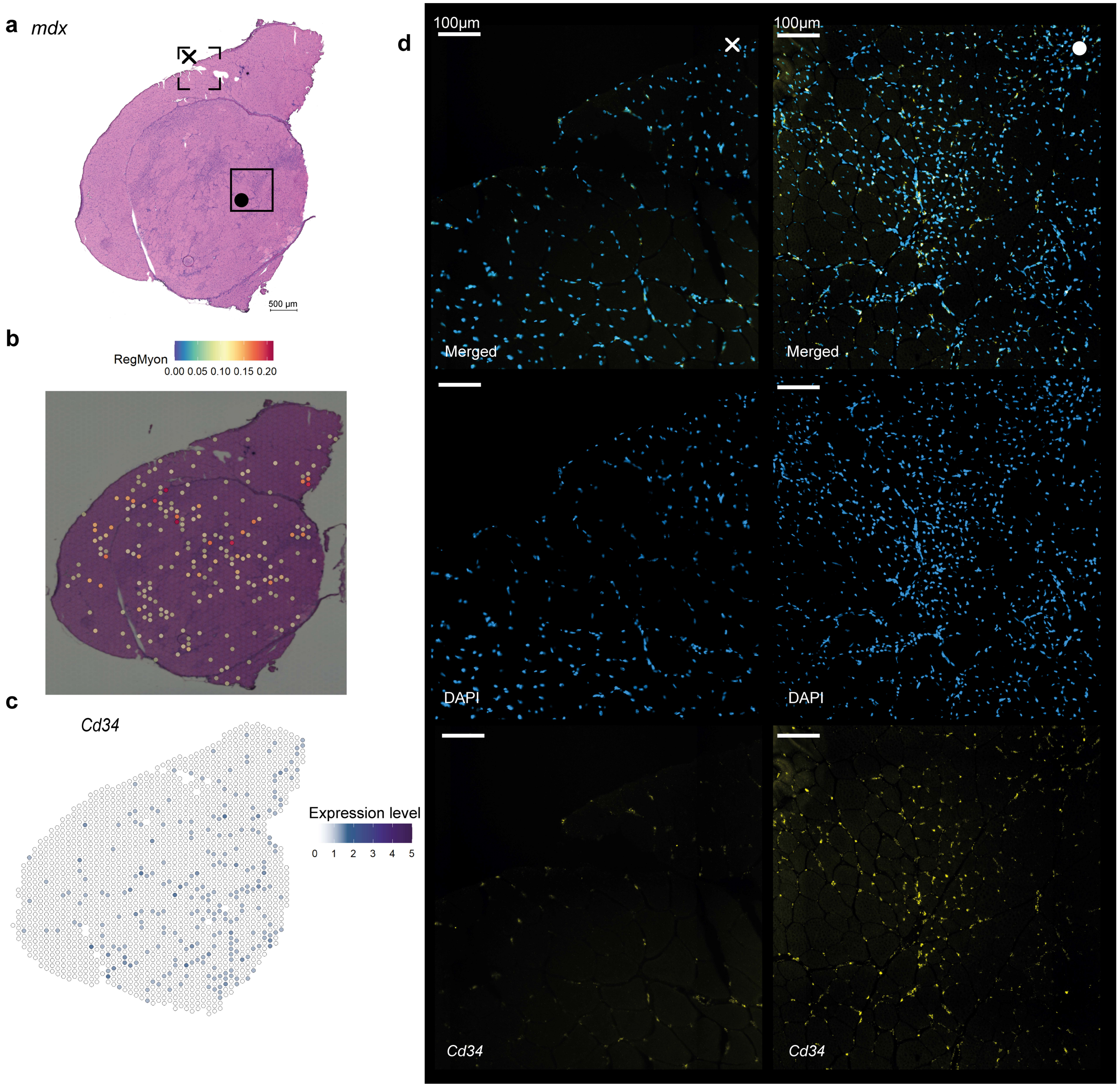

Supplementary Fig. 8. **A comparison of regenerating myonuclei (RegMyon) cells presence between the deconvolution results** *and smFISH (RNAscope) validation on the mdx model. (a) Mdx model with the indications to the approximate location of zoomed-in smFISH images. (b) Location of RegMyon based on spot deconvolution. (c) Cd34, marker gene for progenitor cells, expression pattern based on Visium data. (d) smFISH results in two regions that confirm the absence (cross icon) and presence (filled dot icon) of RegMyon as was expected based on the spot deconvolution results.*

All scale bars in the immunofluorescent images represent 100µm.

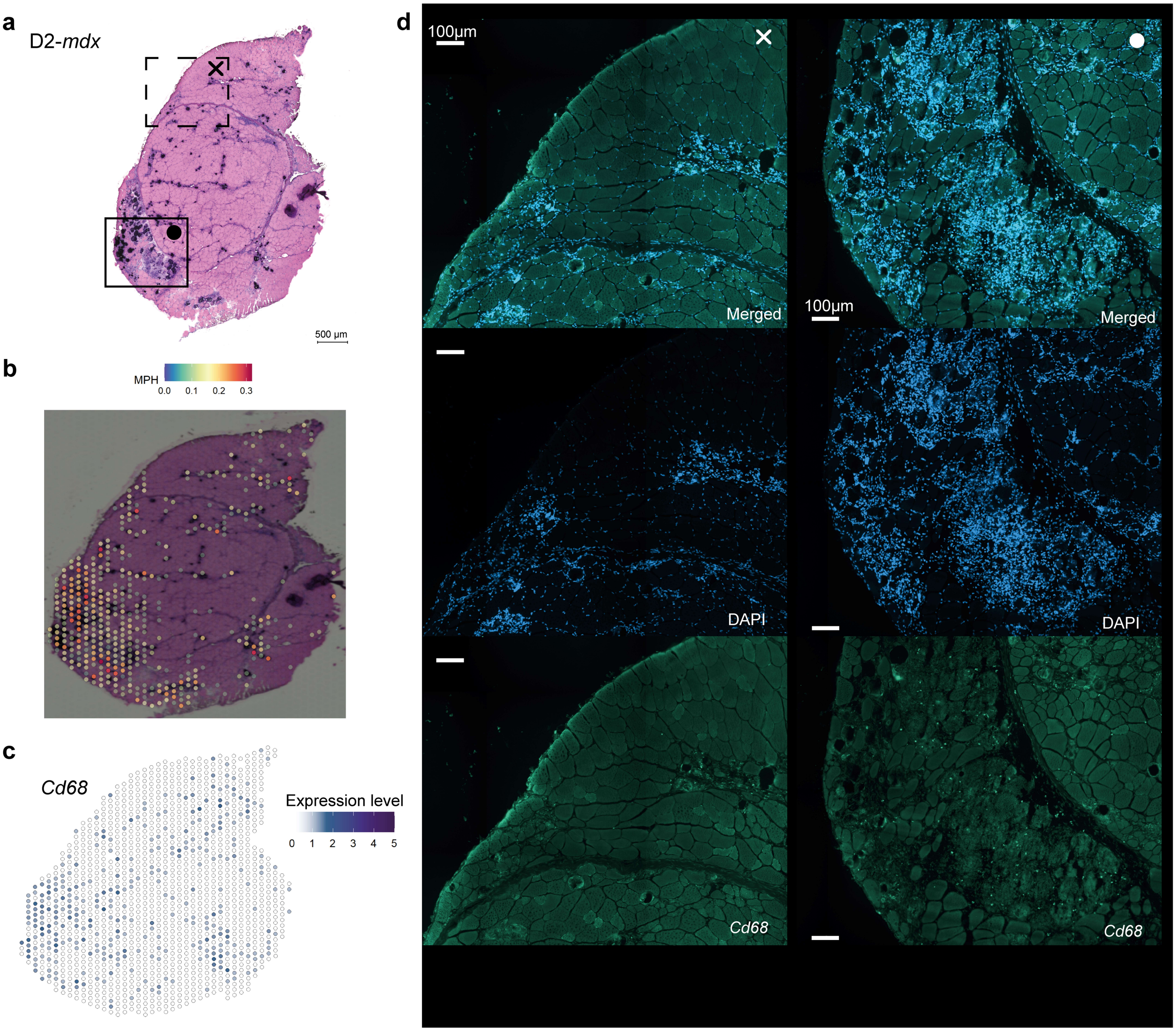

Supplementary Fig. 9. **A comparison of macrophages (MPH) presence between the deconvolution results and smFISH (RNAscope)** *validation on the D2-mdx. (a) D2-mdx model with the indications to the approximate location of zoomed-in smFISH images. (b) Location of MPH based on spot deconvolution. (c) Cd68, marker gene for a subtype of MPH, expression pattern based on Visium data. (d) smFISH results in two regions that confirm the absence (cross icon) and presence (filled dot icon) of MPH as was expected, however in lesser extent, based on the spot deconvolution results.*

All scale bars in the immunofluorescent images represent 100µm.

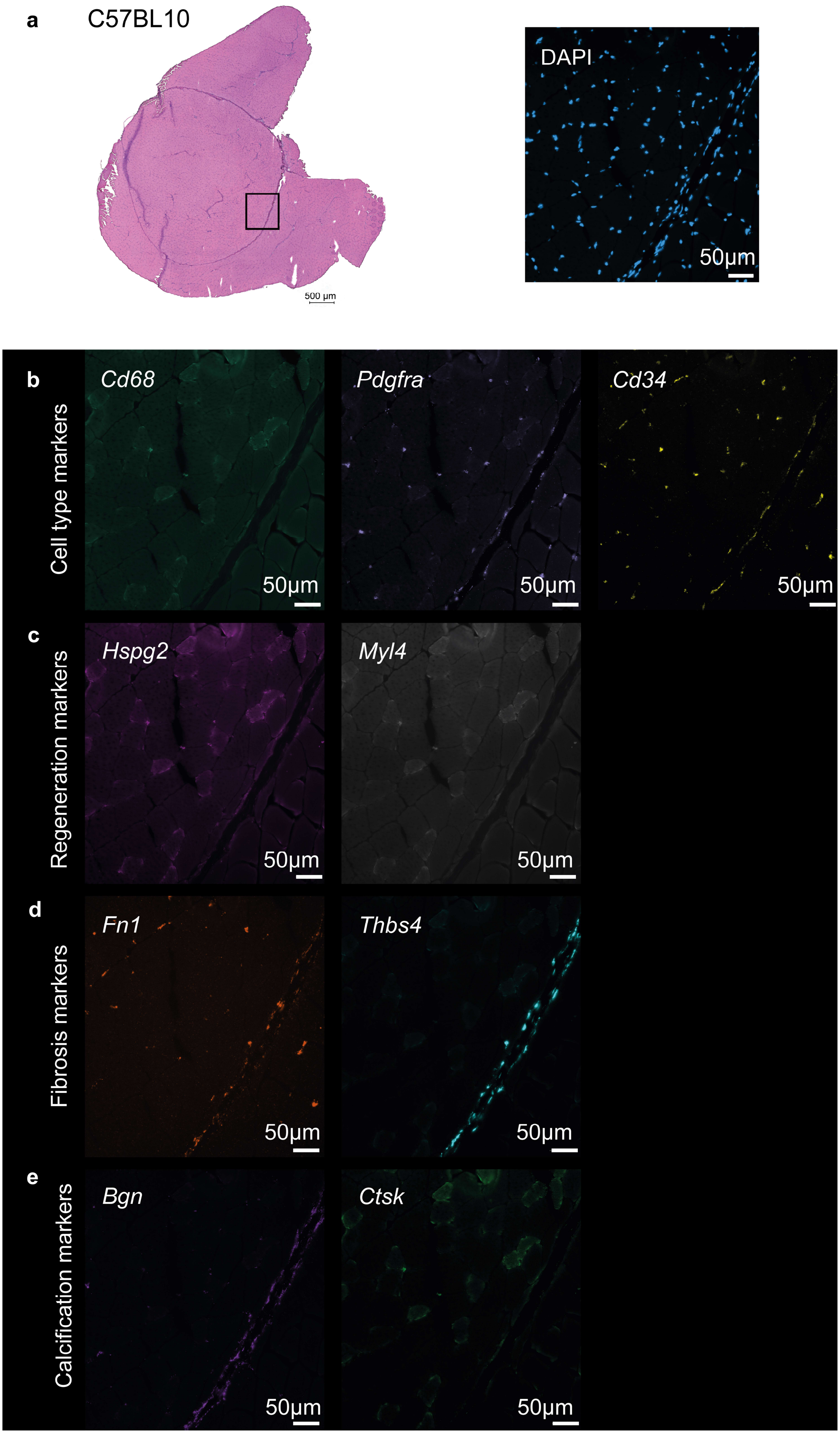

Supplementary Fig. 10. **smFISH results on a consecutive section from the C57BL10 Visium sample.** *(a) Visualized area of the smFISH experiment matches the indicated location in the HE-stained Visium section. (b) Expression of cell type markers: Cd68 (macrophages), Pdgfra (FAPs) and Cd34 (progenitor cells). (c) Identified markers of regeneration (Hspg2, Myl4), (d) fibrosis (Fn1, Thbs4) and (e) calcification (Bgn, Ctsk).*

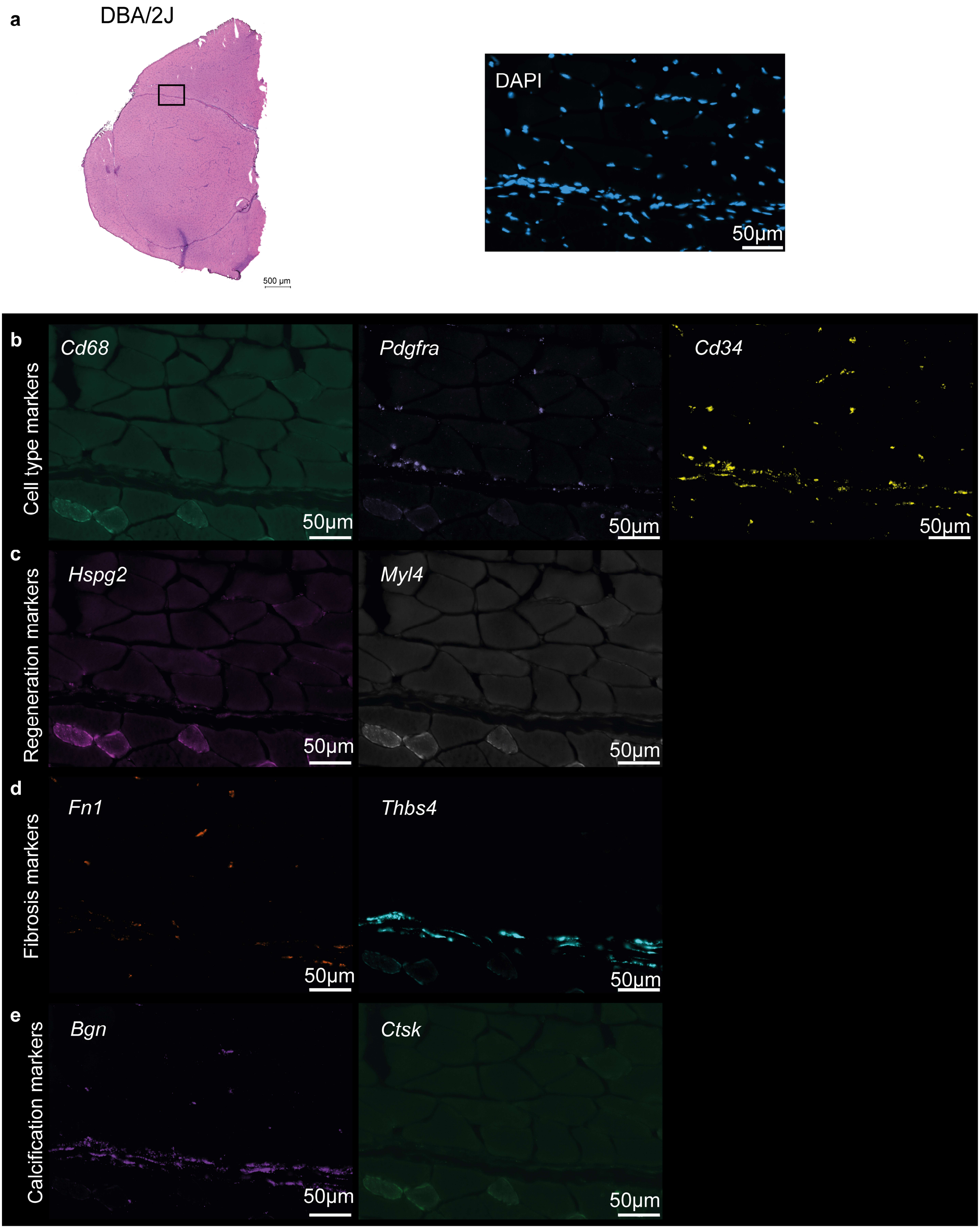

Supplementary Fig. 11. **smFISH results on a consecutive section from the DBA/2J Visium sample.** *(a) Visualized area of the smFISH experiment matches the indicated location in the HE-stained Visium section. (b) Expression of cell type markers: Cd68 (macrophages), Pdgfra (FAPs) and Cd34 (progenitor cells) show no expression of Cd68 and little expression of Pdgfra and Cd34 in and surrounding the connective tissue sheet. (c) Expression of identified markers of regeneration (Hspg2, Myl4), (d) fibrosis (Fn1, Thbs4) and (e) calcification (Bgn, Ctsk) was limited to low expression levels for Thbs4, Bgn and Fn1 in the connective tissue sheet.*

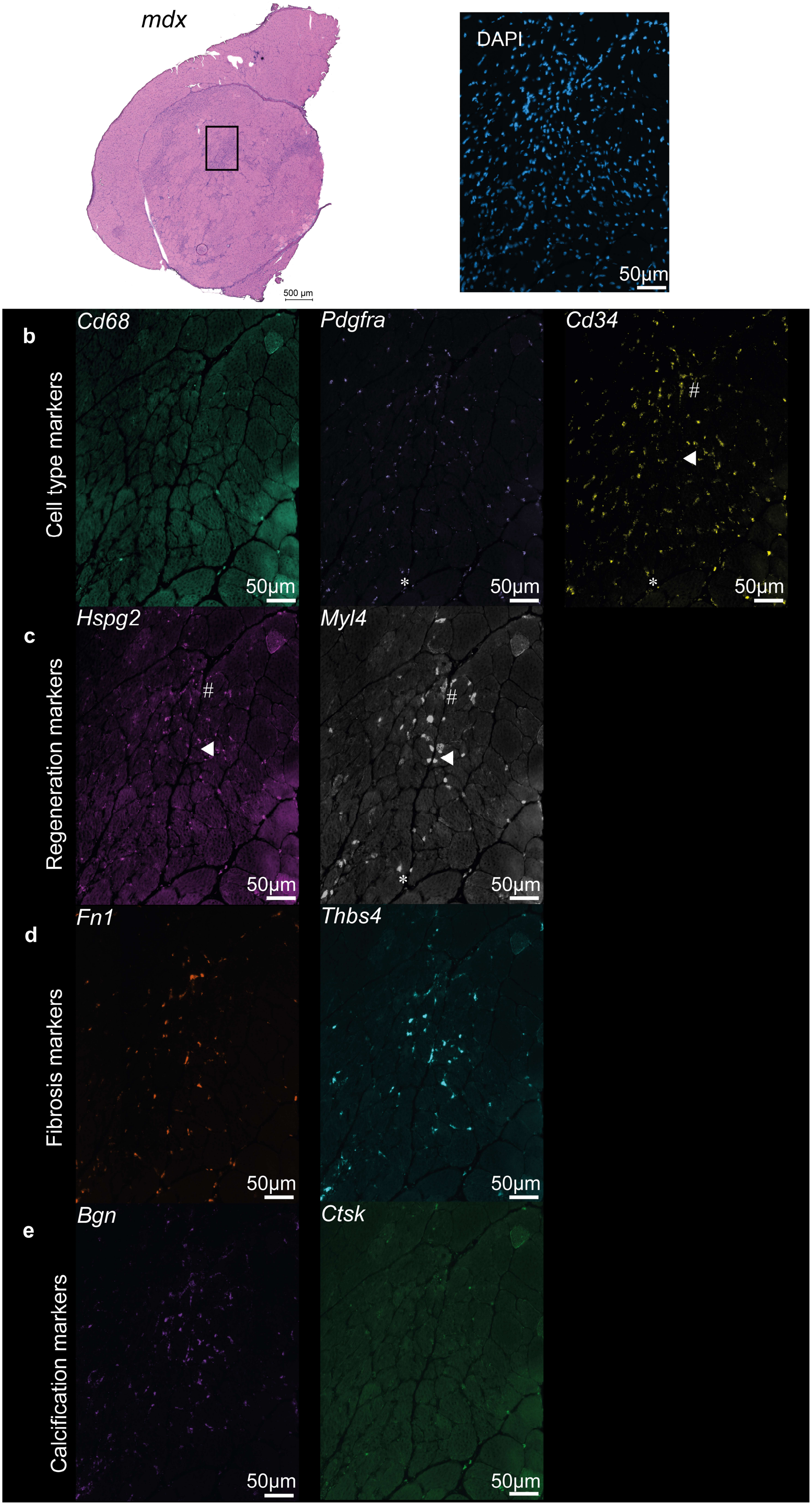

Supplementary Fig. 12. **smFISH results on a consecutive section from the *mdx* Visium sample.** *(a) Visualized area of the smFISH experiment matches the indicated location in the HE-stained Visium section. (b) Expression of cell type markers: Cd68 (macrophages), Pdgfra (FAPs) and Cd34 (progenitor cells). (c) Identified markers of regeneration (Hspg2, Myl4), (d) fibrosis (Fn1, Thbs4) and (e) calcification (Bgn, Ctsk). Highlighted areas of interest show: co-expression of Pdgfra, Cd34 and Myl4 (asterix icon), co-expression of Cd34, Hspg2 and Myl4 (hashtag icon) and co-expression of Hspg2 and Myl4, but no clear co-expression with cell type marker Cd34 (filled triangle icon).*

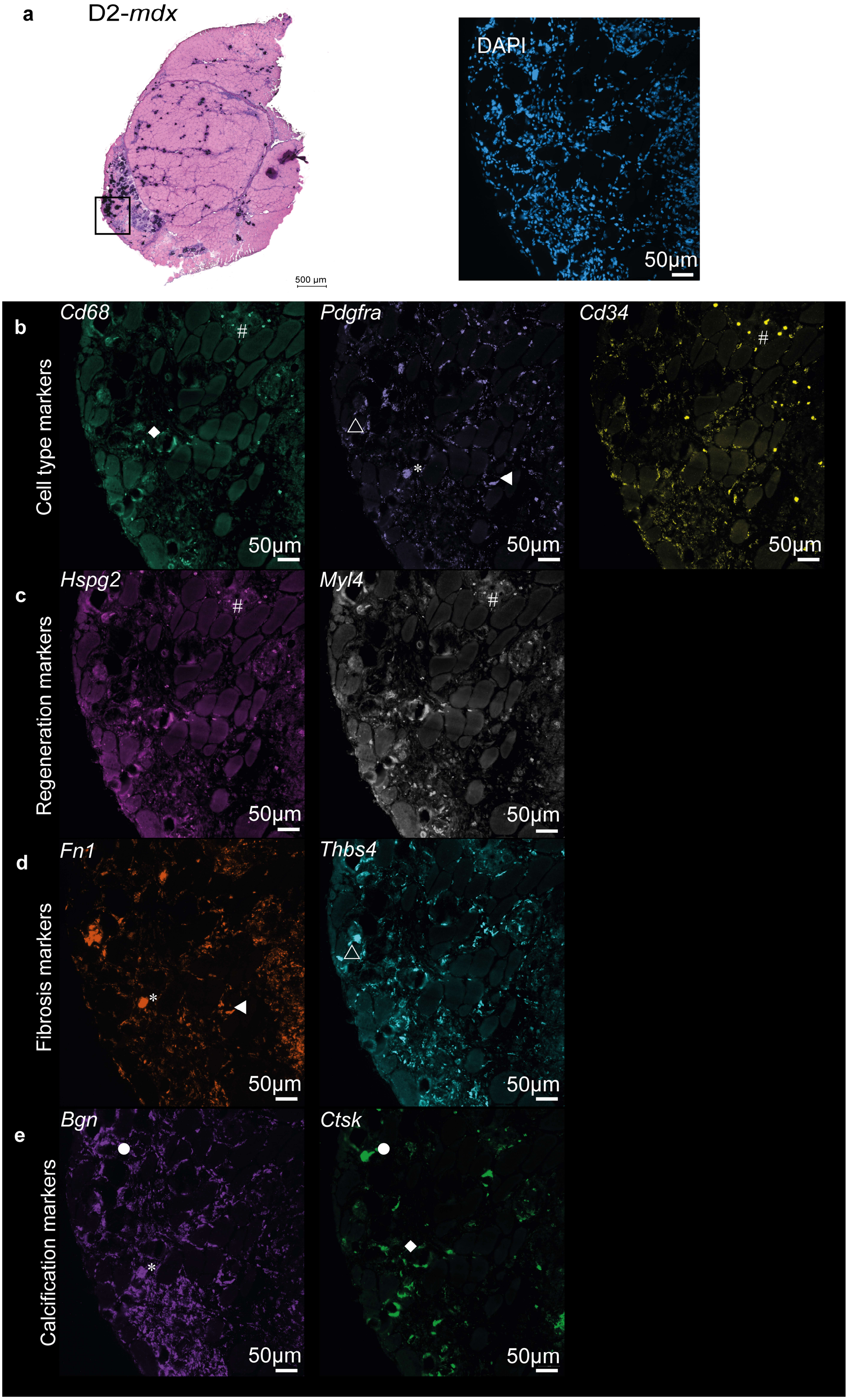

Supplementary Fig. 13. **smFISH results on a consecutive section from the D2-*mdx* Visium sample.** *(a) Visualized area of the smFISH experiment matches the indicated location in the HE-stained Visium section. (b) Expression of cell type markers: Cd68 (macrophages), Pdgfra (FAPs) and Cd34 (progenitor cells). (c) Identified markers of regeneration (Hspg2, Myl4), (d) fibrosis (Fn1, Thbs4) and (e) calcification (Bgn, Ctsk). Highlighted areas of interest show: co-expression of Cd68, Cd34, Hspg2 and Myl4 (hashtag icon), Pdgfra and Thbs4 co-expression (open triangle icon), co-expression of Pdgfra and Fn1 (filled triangle and asterix icons), Cd68 and Ctsk co-expression (filled diamond icon) and an example of Ctsk expression inside the calcified fiber with Bgn expression surrounding the calcified fiber (filled dot icon).*

a

Absolute number of + spots

	C57BL10	*mdx*
**Total no. spots**	2341	1880
Myl4+	122	337
Hspg2+	487	272
**Myl4+/*Hspg2+***	62	310

Percentage of + spots

	C57BL10	*mdx*
**Total no. spots**	2341	1880
Myl4+	5.21	17.93
Hspg2+	20.80	14.47
Myl4+/Hspg2+	2.65	16.49

b

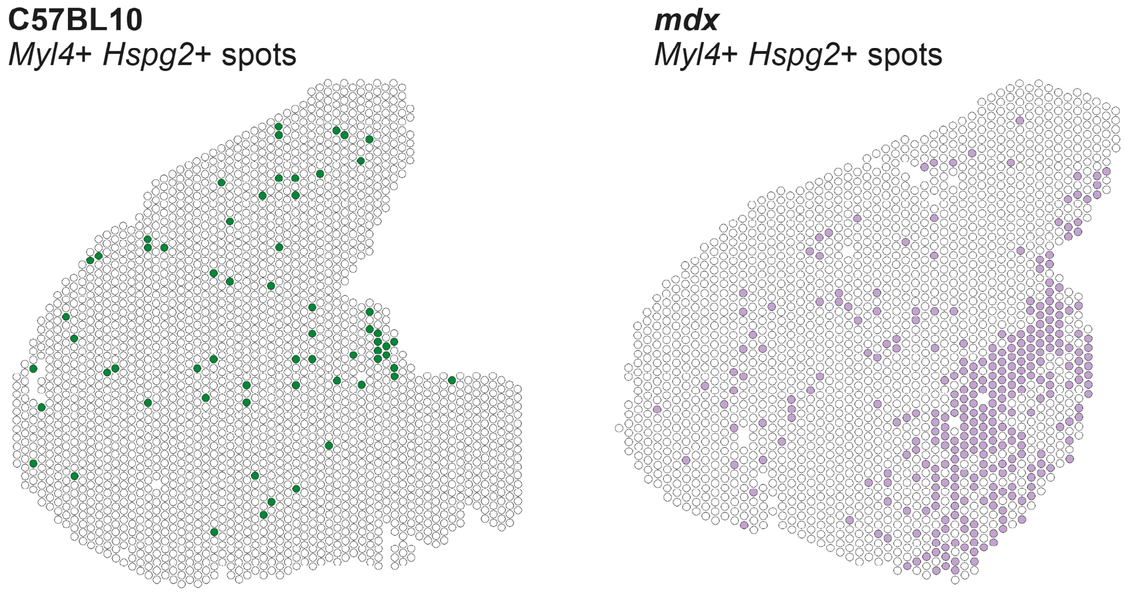

Supplementary Fig. 14. **Co-expression of identified regeneration markers Myl4 and Hspg2.** *(a) The absolute number of spots that are expressing Myl4, Hspg2 or both as well as the percentage of spots that are expressing Myl4, Hspg2 or both. (b) Spots highlighted that are co-expressing Myl4 and Hspg2 in the C57BL10 and mdx mouse.*

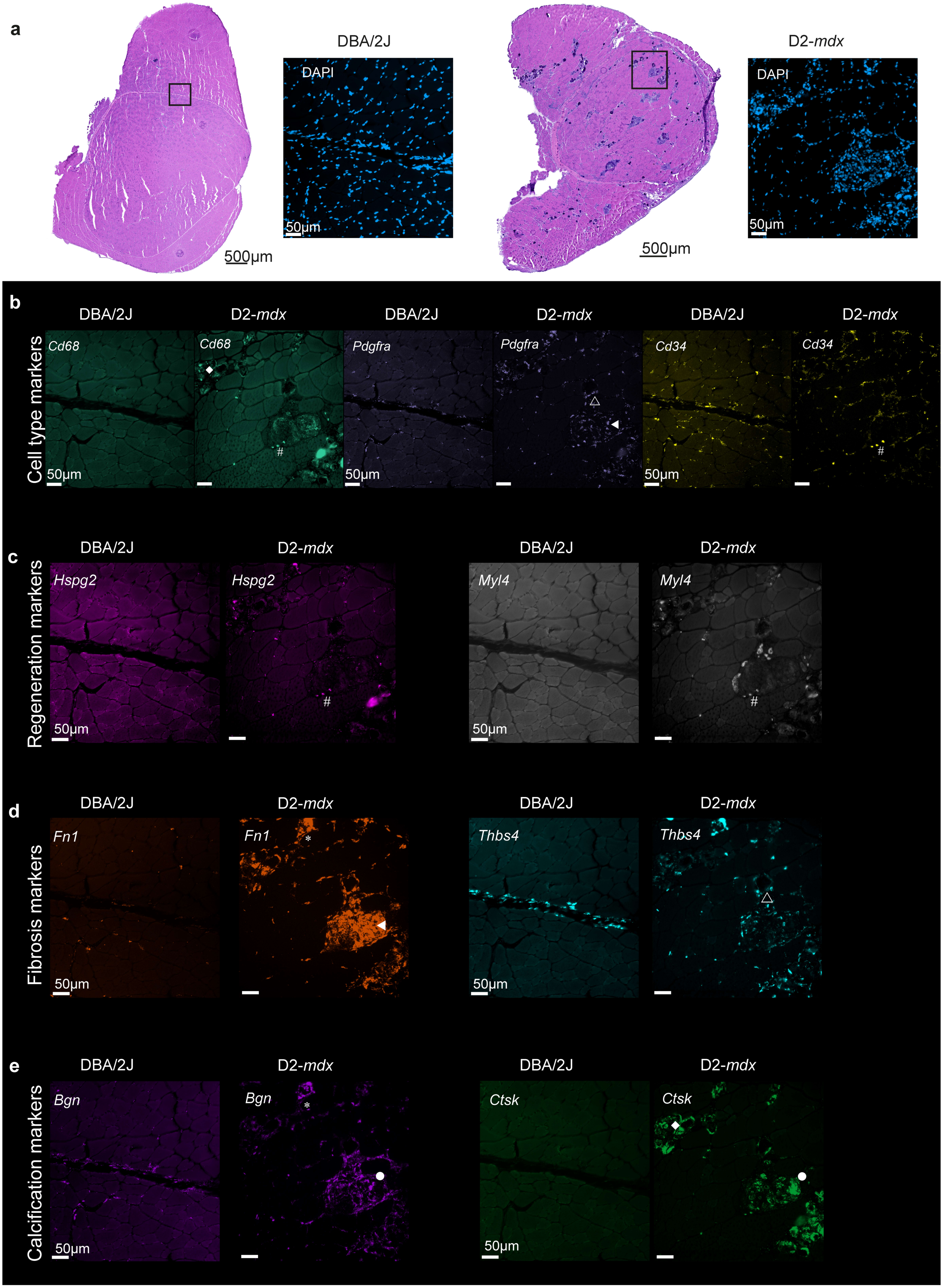

Supplementary Fig. 15. **smFISH results on two independent samples (DBA/2J and D2-*mdx*) as biological validation of the identified genes based on Visium analysis**. *(a) Visualized area of the smFISH experiment matches the indicated areas in the HE-stained sections. (b) Expression of cell type markers: Cd68 (macrophages), Pdgfra (FAPs) and Cd34 (progenitor cells). (c) Identified markers of regeneration (Hspg2, Myl4), (d) fibrosis (Fn1, Thbs4) and (e) calcification (Bgn, Ctsk). Highlighted areas of interest in the D2-mdx mouse model show: co-expression of Cd68, Cd34, Hspg2 and Myl4 (hashtag icon), Pdgfra and Thbs4 co-expression (open triangle icon), co-expression of Pdgfra and Fn1 (filled triangle and asterix icon), Cd68 and Ctsk co-expression (filled diamond icon) and an example of Ctsk expression inside the calcified fiber with Bgn expression surrounding the calcified fiber (filled dot icon). All scale bars in the immunofluorescent images represent 50µm*

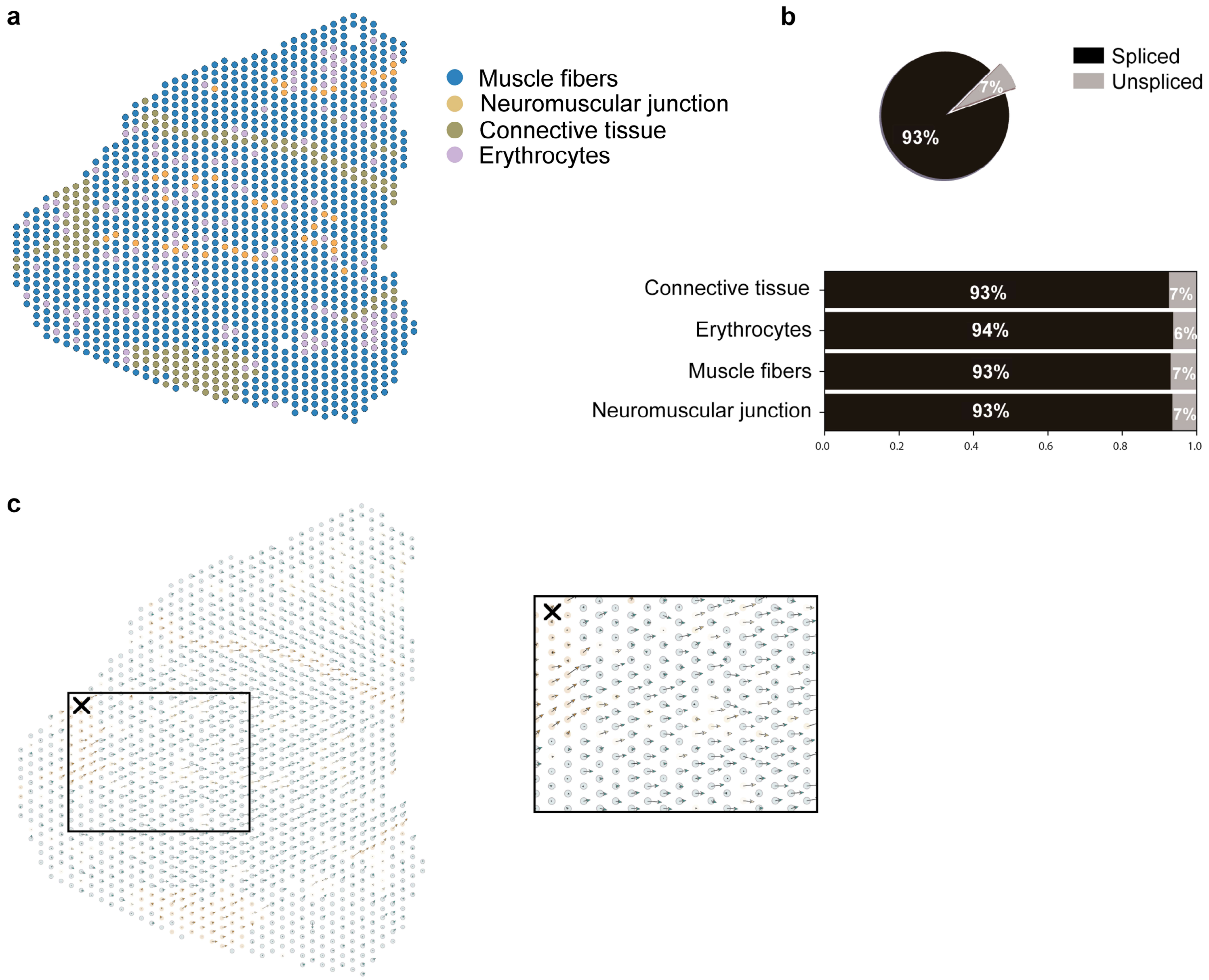

Supplementary Fig. 16. **RNA velocity applied on DBA/2J muscle shows that there are no differentiation dynamics present.** *(a) Annotated clusters of DBA/2J as described before. (b) Proportion of spliced/unspliced counts in the DBA/2J sample and in its annotated clusters (c) Spatial spot-level RNA velocity vectors showing no differentiation pattern as arrows are indicating the direction and strength of the change in transcriptional state in each spot (box with cross for zoomed-in view).*

Supplementary Table 1. Imaging settings used on the ZEISS Axio Scan.Z1 for NMJ staining

	**Channel 1 - BTX**	**Channel 2 - Laminin**	**Channel 3 - DAPI**
Excitation wavelength	493	752	353
Emission wavelength	517	779	465
Light source intensity	50%	65%	50%
Illumination wavelength	450-488	720-750	370-400
Imaging device	OrcaFlash	OrcaFlash	OrcaFlash
Exposure time	150 ms	80 ms	80 ms
Depth of focus	1.62µm	2.44µm	1.45µm

Supplementary Table 2. Product information smFISH HiPlex experiment

**Probe**	**Product code**	**Lot number**	**Channel**
Mm-Myl4-T1	443801-T1	22143A	AF488
Mm-Thbs4-T2	526821-T2	22145A	Dy550
Mm-Bgn-T3	455361-T3	22143A	Dy650
Mm-Fn1-T4	316951-T4	22137A	AF750
Mm-Hspg2-T5	493051-T5	22145A	AF488
Mm-Ctsk-T6	464071-T6	22145A	Dy550
Mm-Pdgfra-T7	480661-T7	22145A	Dy650
Mm-CD34-T8	319161-T8	22145A	AF750
Mm-Cd68-T9	316611-T9	22137A	AF488
HiPlex 12 Positive control probes Mm v2	324433	22090A	
lHiPlex 12 Negative control probe Mm v2	324341	21364A	
HiPlex 12 detection kit Mm v2	324440 324400	2014739	

Supplementary Table 3. Imaging settings used on the ZEISS Axio Scan.Z1 for smFISH HiPlex experiment

	**Channel 1 - DAPI**	**Channel 2 – AF488**	**Channel 3 – AF750**	**Channel 4 – Dy650**	**Channel 5 – Dy547**
**Excitation wavelength**	353	493	752	654	557
**Emission wavelength**	465	517	779	675	574
**Light source intensity**	45%	60%	100%	100%	100%
**Illumination wavelength**	370-400	450-488	720-750	615-648	540-570
**Imaging device**	OrcaFlash	OrcaFlash	OrcaFlash	OrcaFlash	OrcaFlash
**Exposure time**	65 ms	150 ms	150 ms	150 ms	150 ms
**Depth of focus**	1.45µm	1.62µm	2.44µm	2.11µm	1.79µm
