# Supplementary material for: Spatial transcriptomics reveal markers of histopathological changes in Duchenne muscular dystrophy mouse models
Source: Nat Commun. 2023 Aug 15;14:4909. doi: 10.1038/s41467-023-40555-9 (PMC10427630; doi:10.1038/s41467-023-40555-9)
Supplement: Supplementary file 3 — Description of Additional Supplementary Files [file 41467_2023_40555_MOESM3_ESM.pdf]

Description of Additional Supplementary Files

Supplementary Data File 1. List of marker genes per cluster for each mouse model.

Supplementary Data File 2. List of marker genes for the muscle fibers cluster.

Supplementary Data File 3. List of differentially expressed genes.
