# Supplementary material for: Spatial transcriptomics reveal markers of histopathological changes in Duchenne muscular dystrophy mouse models
Source: Nat Commun. 2023 Aug 15;14:4909. doi: 10.1038/s41467-023-40555-9 (PMC10427630; doi:10.1038/s41467-023-40555-9)
Supplement: Supplementary file 7 — Reporting Summary [file 41467_2023_40555_MOESM7_ESM.pdf]

nature portfolio

Corresponding author(s): Pietro Spitali

Last updated by author(s): Jul 4, 2023

Reporting Summary

Nature Portfolio wishes to improve the reproducibility of the work that we publish. This form provides structure for consistency and transparency in reporting. For further information on Nature Portfolio policies, see our Editorial Policies and the Editorial Policy Checklist.

Statistics

For all statistical analyses, confirm that the following items are present in the figure legend, table legend, main text, or Methods section.

n/a	Confirmed	
☐	☒	The exact sample size (*n*) for each experimental group/condition, given as a discrete number and unit of measurement
☐	☒	A statement on whether measurements were taken from distinct samples or whether the same sample was measured repeatedly
☐	☒	The statistical test(s) used AND whether they are one- or two-sided<br>*Only common tests should be described solely by name; describe more complex techniques in the Methods section.*
☒	☐	A description of all covariates tested
☒	☐	A description of any assumptions or corrections, such as tests of normality and adjustment for multiple comparisons
☐	☒	A full description of the statistical parameters including central tendency (e.g. means) or other basic estimates (e.g. regression coefficient) AND variation (e.g. standard deviation) or associated estimates of uncertainty (e.g. confidence intervals)
☐	☒	For null hypothesis testing, the test statistic (e.g. *F*, *t*, *r*) with confidence intervals, effect sizes, degrees of freedom and *P* value noted<br>*Give P values as exact values whenever suitable.*
☒	☐	For Bayesian analysis, information on the choice of priors and Markov chain Monte Carlo settings
☒	☐	For hierarchical and complex designs, identification of the appropriate level for tests and full reporting of outcomes
☒	☐	Estimates of effect sizes (e.g. Cohen's *d*, Pearson's *r*), indicating how they were calculated

Our web collection on statistics for biologists contains articles on many of the points above.

Software and code

Policy information about availability of computer code

Data collection	There was no software used to obtain data. Data was collected by performing a spatial transcriptomics experiment (Visium) and open source datasets were used for specific analysis.
Data analysis	Raw sequencing data was processed through 10xGenomics Space Ranger (v1.1.0)pipelines mkfastq and count. The output data files of these pipelines were inputted into R (version 4.1.1) for further analyses of which most were done using the Seurat package (version 4.0.5). Specific code that was used will be made available on GitHub. Normalization was performed with SCTransform. Hereafter, Principal Component Analysis was applied (RunPCA), and the top 30 principal components were used to generate a neighborhood graph using 20 neighbors (FindNeighbors). Next, data was clustered (FindClusters) with different resolutions (C57BL10 = 0.4, DBA/2J = 0.8, mdx = 0.8, D2-mdx = 0.4) and UMAP embedding was generated (RunUMAP). The FindAllMarkers (min.pct = 0.1, only.pos = FALSE) function was used to identify cluster-specific marker genes. The top 20 markers per cluster genes, based on Bonferroni-corrected p-values, were exported to EnrichR. Here, we looked at enrichment in cell types in the PanglaoDB Augmented 2021 reference dataset to guide the annotation of the clusters. To deconvolute the spots in the spatial dataset, SPOTlight was used. To identify differentially expressed genes, the FindMarkers function in Seurat was applied. Finally, we used kallisto/BUStools, to map the sequencing reads (fastq) to the reference genome (mm10) while quantifying intronic (unspliced) and exonic (spliced) reads. Next, we applied the RNA velocity pipeline (dynamic model implemented in the scvelo 0.2.3 python (v.3.8.5) package) to estimate the RNA velocity vector (i.e differentiation) of each spot in its spatial context. The codes used for the analysis in this manuscript are available on GitHub: https://github.com/lauraheezen/SpatialAnalysis_DMD_mice.git . This code can be cited with the following DOI identifier: 10.5281/zenodo.8073645.

For manuscripts utilizing custom algorithms or software that are central to the research but not yet described in published literature, software must be made available to editors and reviewers. We strongly encourage code deposition in a community repository (e.g. GitHub). See the Nature Portfolio guidelines for submitting code & software for further information.

Data

Policy information about availability of data

All manuscripts must include a data availability statement. This statement should provide the following information, where applicable:

- Accession codes, unique identifiers, or web links for publicly available datasets
- A description of any restrictions on data availability
- For clinical datasets or third party data, please ensure that the statement adheres to our policy

All sequencing data (fastq files) and data output files that were produced through 10xGenomics spaceranger pipelines mkfastq and count. Raw data files are available in Gene Expression Omnibus (GEO) under accession number GSE199659. Processed data files are made available on Zenodo under DOI: 10.5281/zenodo.7401196. The PanglaoDB dataset (doi:10.1093/database/baz046) was used for gene enrichment analysis through EnrichR in guidance towards cluster annotation.

Field-specific reporting

Please select the one below that is the best fit for your research. If you are not sure, read the appropriate sections before making your selection.

☒ Life sciences ☐ Behavioural & social sciences ☐ Ecological, evolutionary & environmental sciences

For a reference copy of the document with all sections, see nature.com/documents/nr-reporting-summary-flat.pdf

Life sciences study design

All studies must disclose on these points even when the disclosure is negative.

Sample size	There was no sample size calculation performed prior to the experiment. A total of n=1 for each strain was included for this proof-of-principle Visium experiment, but an additional n=3 mice per strain were euthanized for validation experiments using smFISH (RNAscope) which is a highly sensitive method to capture the spatial pattern of RNA expression of target genes. To have a total of n=4 for these kind of experiments is widely accepted. These numbers were chosen based on previous published work using the visium spatial technology for other non-muscular diseases.
Data exclusions	Mitochondrial reads were excluded from data analysis as they are known to introduce a bit of bias into the data and were not of interest in our analysis. The following genes were excluded: mt-Nd1, mt-Nd2, mt-Co1, mt-Co2, mt-Atp8, mt-Atp6, mt-Co3, mt-Nd3, mt-Nd4l, mt-Nd4, mt-Nd5, mt-Nd6, mt-Cytb. Furthermore, spots with very few counts or extremely high counts were excluded by filtering each sample separately based on the number of UMIs (nCount) and the number of genes (nFeature). The cut-offs were determined by visual inspection of the violin plots for each sample: for C57BL10 nCount >= 150 and <= 40000, nFeature >=150 and <=5000, for DBA/2J nCount >= 100 and <= 20000, nFeature >=200 and <=4000, for mdx nCount >= 200 and <= 40000, nFeature >=250 and <=5000 and for D2-mdx nCount >= 100 and <= 20000, nFeature >=150 and <=5000.
Replication	Data can be replicated by using the R code that was used for performing the analyses and inputting the data that is available on GEO. The codes used for the analysis in this manuscript are available on GitHub: https://github.com/lauraheezen/SpatialAnalysis_DMD_mice.git
Randomization	Randomization was not relevant for this study as it was an n=1 per mouse model that was included.
Blinding	Again, blinding was not relevant for this study as n=1 per mouse strain was included and there were no analysis done that are based on manual scoring by the researcher or leave room for different interpretation.

Reporting for specific materials, systems and methods

We require information from authors about some types of materials, experimental systems and methods used in many studies. Here, indicate whether each material, system or method listed is relevant to your study. If you are not sure if a list item applies to your research, read the appropriate section before selecting a response.

Materials & experimental systems

n/a	Involved in the study	
☐	☒	Antibodies
☒	☐	Eukaryotic cell lines
☒	☐	Palaeontology and archaeology
☐	☒	Animals and other organisms
☒	☐	Human research participants
☒	☐	Clinical data
☒	☐	Dual use research of concern

Methods

n/a	Involved in the study	
☒	☐	ChIP-seq
☒	☐	Flow cytometry
☒	☐	MRI-based neuroimaging

Antibodies

Antibodies used	Primary antibody: Rabbit-anti-laminin (Abcam, ab11575, Lot GR233309-2) - Secondary antibody 1 750 (Abcam, goat-anti-rabbit 750, ab175735; Lot GR148346—6, 1:500 dilution) - Secondary antibody 2: BTX-488 (donkey-anti-human-IgG 488 with BTX, Invitrogen, Thermo Fisher Scientific, OR, USA, B13422, Lot 2194175, 1:500 dilution)<br>Mounting medium: Prolong Gold antifade reagent with DAPI (Invitrogen, Thermo Fischer Scientific, Eugene, OR, USA, P36935, Lot 2465351)
Validation	From the Abcam website - Abcam, ab11575<br>Description<br>Rabbit polyclonal to Laminin<br><br>Host species<br>Rabbit<br>Specificity<br>In dot blot immunoassay this antibody does not react with Fibronectin, Vitronectin, Collagen IV, or Chondroitin sulfate types A, B, and C.<br><br>Tested applications<br>Suitable for: Dot blot, IHC-Pmore details<br>Species reactivity<br>Reacts with: Mouse, Human<br>Predicted to work with: Rat, Horse, Dog, Pig, Xenopus laevis, Reptile, Mammals, Amphibian<br><br>Immunogen<br>Full length native protein (purified) corresponding to Laminin. The immunogen is laminin isolated from the basement membrane of Englebreth Holm-Swarm (EHS) mouse sarcoma.

Animals and other organisms

Policy information about studies involving animals; ARRIVE guidelines recommended for reporting animal research

Laboratory animals	10 week old male mice were used in this study of the following strains (n=4 per strain): C57BL/10ScSn-Dmdmdx/J (mdx), C57BL/10ScSnJ (C57BL10), D2.B10-Dmdmdx/J (D2-mdx) and DBA/2J (DBA/2J). Mice were housed in individually ventilated cages (lights on/off 0700/1900h) at 20.5 centigrate degrees, humidity between 40% and 70%, with ad libitum access to water and chow.
Wild animals	The study did not involve wild animals
Field-collected samples	The study did not include field-collected samples
Ethics oversight	Mice were handled according to the guidelines established by the Animal Experiment Committee (Dierexperimenten commissie) of the Leiden University Medical Center (protocol PE.17.246.026).

Note that full information on the approval of the study protocol must also be provided in the manuscript.
